# Neuroticism is related to functional outcomes after surgically treated proximal humerus fractures

**DOI:** 10.1016/j.jseint.2025.02.018

**Published:** 2025-03-26

**Authors:** Denise E. de Gruijter, Leanne S. Blaas, Kelly van Winden, Rosa E. Boeschoten, Susan van Dieren, Michel P.J. van den Bekerom, Robert Jan Derksen

**Affiliations:** a Department of Trauma Surgery, Zaandam Medical Center, Zaandam, The Netherlands; b Department of Psychiatry, Amsterdam University Medical Center, Amsterdam, The Netherlands; c Department of Orthopedic Surgery, OLVG, Amsterdam, The Netherlands

**Keywords:** Proximal humerus fracture, Shoulder, Neuroticism, Personality, Surgery, Functional outcomes

## Abstract

**Background:**

It is known that physical factors play an important role in the outcome after a proximal humerus fracture (PHF). However, an increasing body of evidence shows that psychological factors are of importance as well. As such, it follows that the level of neuroticism in a patient might be especially important. This study aims to examine the correlation between neuroticism levels and functional outcomes following surgically treated PHFs.

**Methods:**

This is a retrospective cohort analysis. Patients with PHFs who were surgically treated from 2013 to 2023 and had a minimum 1-year follow-up were eligible. During follow-up, shoulder range of motion and functional scores (Constant Shoulder Score [CSS], Oxford Shoulder Score, and quick Disability of the Arm, Shoulder and Hand) were measured. To ascertain the level of neuroticism, the short-revised version of the Eysenck Personality Questionnaire was administered.

**Results:**

In total, 65 patients were included in the study. Eighty-nine percent of included subjects were women (n = 58) and the median age was 72.3 ± 7.4 years. Seventy-four percent (n = 48) was treated with a reverse total shoulder arthroplasty and 60% (n = 39) was operated on their dominant side. The level of neuroticism is correlated with the CSS for fracture side (r = −0.28; *P* = .023), correlated with the Oxford Shoulder Score (r = −0.46; *P* < .001) and to the quick Disability of the Arm, Shoulder and Hand (r = 0.30; *P* = .017). The CSS difference score between the fracture side and the nonaffected side was not correlated with the level of neuroticism (r = 0.20; *P* = .12).

**Conclusion:**

A higher level of neuroticism is related to worse functional outcomes after a surgically treated PHF.

Shoulder injuries are among the most common injuries presented on the emergency department.^13^^,^^22^ Physical factors, such as age, sex, preoperative mobility, strength, and pre-existing comorbidities,^1^^,^^17^^,^^27^ play an important role in the type of treatment, rehabilitation process and outcomes after proximal humerus fracture (PHF). However, psychological factors need to be considered as well. Studies examining the relationship between psychosocial factors and mental vulnerability on the outcomes after elective shoulder surgery, in general, have shown negative correlations.^6^^,^^15^^,^^24^^,^^30^^,^^31^ It is reported that low resilience, negative thoughts, tendency for catastrophizing and/or signs of depression or anxiety have a negative relation on functional outcomes after joint and fracture surgery.^5^^,^^6^^,^^15^^,^^21^^,^^23^^,^^30^, ^31^, ^32^, ^33^ Mental vulnerability is linked to the personality trait neuroticism and can be characterized by the tendency to experience negative affect, especially when threatened, frustrated, or facing loss.^25^ Neuroticism is also the personality trait shown to be the most important concerning worse outcomes of physical health and disease in patients.^11^^,^^35^

### Rationale

Although psychological factors are associated with worse outcomes after joint arthroplasty,^5^^,^^15^^,^^32^^,^^33^ this has not yet been assessed in surgical treatment of PHFs. Patients with fractures are expected to be less capable of mentally preparing for surgery since sustaining a fracture is an acute event. Generally, patients with fractures are operated on within a 2- to 3-week time frame, especially when open reduction and internal fixation is performed. Consequently, patients with fractures suddenly face a significant life change that demands resilience, flexibility, and positivity. The psychological impact might be quite different compared with patients undergoing arthroplasty for osteoarthritis who have more time to cope with progressive chronic symptoms ultimately leading to an operation. Therefore, patients with a higher level of neuroticism are expected to struggle more to adjust, rendering our hypothesis that worse functional scores are expected in these patients.

### Purpose

The primary aim of this study is to determine whether neuroticism is related to functional outcomes following surgical treatment of PHFs.

## Materials and methods

### Study design

This study is a retrospective cohort analysis. Patients were eligible to participate in the study if they had a PHF that was surgically treated (either open reduction and internal fixation or reverse total shoulder arthroplasty) between 2013 and 2023, were 18 years or older, and had completed at least a 1-year follow-up during which functional questionnaires were completed.

Patients who had additional fractures (polytrauma), patients who underwent a second surgery for a complication or had a pathological fracture before the 1-year follow-up, were excluded from the study.

When the patients were eligible, study information was sent to them, and they were called to ask if they wanted to participate. After receiving informed consent, the short-revised version of the Eysenck Personality Questionnaire (EPQ-RSS)^2^ assessing the level of neuroticism was administered over the phone by an independent clinician-researcher.

Functional outcome measurements were extracted from our Shoulder Registry. The functional outcomes were measured by an MD researcher between 2013 and 2023 using the Constant Shoulder Score (CSS), Oxford Shoulder Score (OSS) and the quick Disability of the Arm, Shoulder and Hand (qDASH) and tracked in our Shoulder registry.^3^^,^^8^^,^^10^^,^^12^^,^^14^^,^^18^ Baseline characteristics were extracted from the electronic patient records.

### Variables and outcome measures

The aim of this study was to investigate whether neuroticism correlates with functional outcomes following surgery for a PHF. Neuroticism was assessed using the EPQ-RSS questionnaire.^2^ The EPQ-RSS is a questionnaire consisting of 10 items, with a total possible score of 10 points. A higher score indicates a higher level of neuroticism, meaning the patient experiences more anxious and depressive thoughts than a person with low scores and thus a low level of neuroticism. The EPQ-RSS was administered at least 1 year after surgery. To minimize researcher bias, all questionnaires were administered by the same researcher.

The functional outcomes were assessed using three different questionnaires: CSS, OSS, and qDASH. The CSS is a 100-point scale, where a higher score indicates better shoulder function. The CSS is the most objective scale among the functional outcomes, as it includes range of motion in its assessment.^3^^,^^8^^,^^10^ The OSS has a maximum score of 48 points, with higher scores indicating better function. The OSS considers daily activities, sleep quality, and pain in its evaluation.^3^^,^^8^^,^^12^^,^^14^ Lastly, the qDASH is measured on a scale from 0 to 100, where a higher score indicates worse function. The qDASH focuses on the ability to perform activities and pain.^3^^,^^8^^,^^18^ The functional scores were obtained at least 10 months, but on average 12 months, after the surgery.

The range of motion measurements, used for the CSS, were taken during the 1-year follow-up visit (at least 10 months after the surgery), using a goniometer, a reliable measurement tool.^19^

### Statistical analysis

Statistical analysis was done using IBM SPSS statistics version 28 (IBM Corp., Armonk, NY, USA). As the study encompasses assessment of a possible correlation, no power analysis was performed. However, for testing 1 correlation, the advised number of participants is 50, which we achieved.^34^ Descriptive statistics were performed for all patients. Continuous variables (CSS, OSS, and qDASH) are presented as mean with standard deviation for normally distributed data and median with quartiles for nonparametric data.

The correlation between neuroticism and functional outcomes will be tested using the Pearson correlation coefficient for normally distributed data and the Spearman rank correlation for nonparametric data.

We identified and adjusted for potential confounding variables by conducting partial correlation analyses, adjusting for identified confounders (age, side of the fracture/operation side, Neer classification, osteoporosis, diabetes, smoking, and gender) to ensure the robustness of our findings. Multivariate regression analysis was used to compare correlation coefficients and bootstrapping was used for nonparametric data.

## Results

### Descriptive data

Eighty-five patients, on paper, were eligible to participate in the study. Seven patients had died, for 2 patients the phone number was incorrect, and 12 patients did not want to participate. In total, 65 patients were included in the study. Of the patients (n = 58) were women and the median age was 72.3 ± 7.4 (Table I). Seventy-four percent (n = 48) was treated with a reverse total shoulder arthroplasty and 60% (n = 39) was operated on their dominant side. The median follow-up time was 12 months and the mean follow-up was 20 months (Table I).

**Table I:** Demographics of cohort (n = 65).

Variable	Value
Age (yr)	72.3 ± 7.4
Women (n)	89% (58)
Dominant side operated (n)	64% (39)
EPQ-RSS	3.6 ± 2.7
Follow-up time (mo)	12 ± 0.6
Surgery type	
Reverse total shoulder arthroplasty (n)	74% (48)
Plate fixation (n)	25% (17)
Neer classification scores	
1	2% (1)
2	40% (26)
3	42% (27)
4	17% (11)
Functional scores	
CSS difference	20 ± 16
CSS fracture side	72 ± 19
OSS	41 ± 8
qDASH	18.4 ± 15.2

*EPQ-RSS*, Eysenck Personality Questionnaire Revised Short Version; *CSS*, Constant Shoulder Score; *OSS*, Oxford Shoulder Score; *qDASH*, quick Disability of the Arm, Shoulder and Hand; *SD*, standard deviation.

Data presented as mean ± SD or %(n). The follow-up time is presented as median ± SD.

### Results

**Figure 1:**
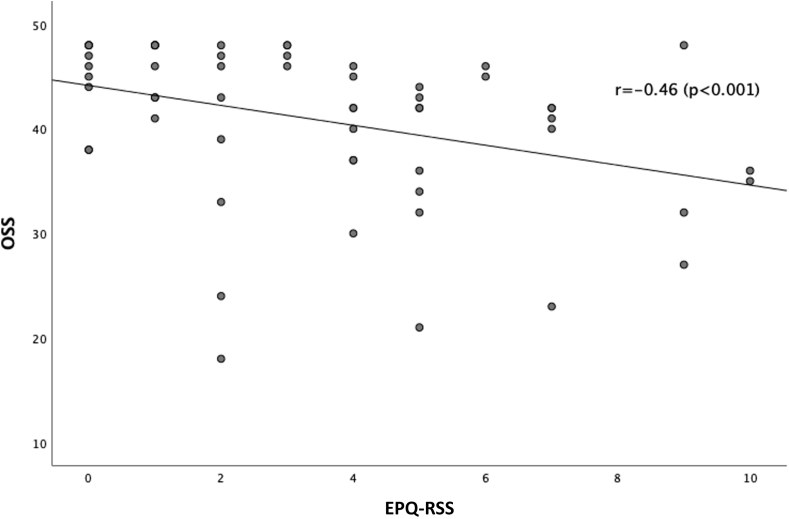
This scatter plot graph depicts the correlation between the OSS at 1 year follow-up and the level of neuroticism (EPQ-RSS). With higher OSS scores indicating better functional outcomes and higher EPQ-RSS scores indicating higher level of neuroticism. The correlation coefficient is shown in the graph. *OSS*, Oxford Shoulder Score; *EPQ-RSS*, Eysenck Personality Questionnaire Revised Short Version.

**Figure 2:**
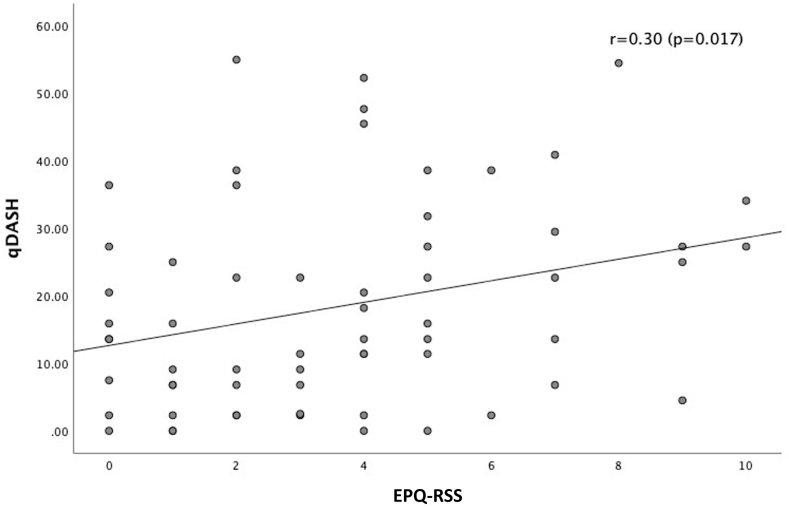
This scatter plot graph depicts the correlation between the qDASH at 1 year follow-up and the level of neuroticism (EPQ-RSS). With lower qDASH scores indicating better functional outcomes and higher EPQ-RSS scores indicating higher level of neuroticism. The correlation coefficient is shown in the graph. *qDASH*, quick Disability of the Arm, Shoulder and Hand; *EPQ-RSS*, Eysenck Personality Questionnaire Revised Short Version.

**Figure 3:**
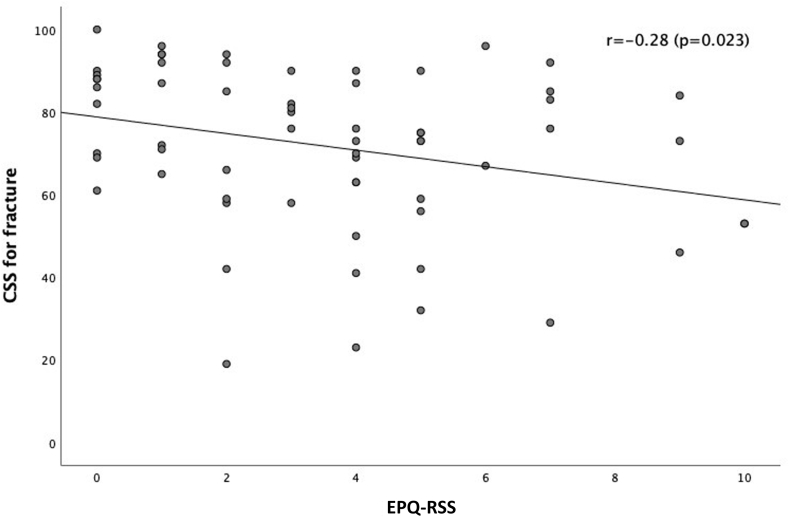
This scatter plot graph depicts the correlation between the CSS for fracture side at 1 year follow-up and the level of neuroticism (EPQ-RSS). With higher CSS for fracture side scores indicating better functional outcomes and higher EPQ-RSS scores indicating higher level of neuroticism. The correlation coefficient is shown in the graph. *CSS*, Constant Shoulder Score; *EPQ-RSS*, Eysenck Personality Questionnaire Revised Short Version.

The level of neuroticism is negatively correlated to the OSS (r = −0.46; *P* < .001) (Fig. 1) and CSS (r = −0.28; *P* = .023) (Fig. 2) for fracture side, indicating that a higher level of neuroticism correlates with worse functional outcome. The level of neuroticism is positively correlated to the qDASH (r = 0.30; *P* = .017), meaning that a higher level of neuroticism correlates with worse functional outcome (Fig. 3). The difference in CSS score between fracture side and nonaffected side was not correlated to the level of neuroticism (r = 0.20; *P* = .12).

Multivariable regression analysis showed no other correlation for the CSS at the fractured side, OSS, or qDASH score (Table II).

**Table II:** *P* values of the multivariable regression analysis.

Variable	CSS for fracture side	OSS	qDASH
Gender	0.333	0.155	0.216
Age	0.624	0.671	0.342
Dominant side = operated side	0.242	0.910	0.492
Neer classification	0.611	0.850	0.332
Surgery type	0.166	0.227	0.106
Diabetes	0.085	0.089	0.757
Osteoporosis	0.248	0.284	0.969

*CSS*, Constant Shoulder Score; *OSS*, Oxford Shoulder Score; *qDASH*, quick Disability of the Arm, Shoulder and Hand.

## Discussion

Psychological factors play an important role in the sort of treatment, rehabilitation and outcome after a PHF. Previous studies have shown that neuroticism (tendency toward anxiety, negative affect, depression, and low resilience) is linked to being an important trait concerning worse outcomes after joint surgery. This led us to anticipate worse functional outcomes for patients with higher neuroticism scores.

Our study has demonstrated that having neuroticism as the main personality trait is associated with poorer functional outcomes across all 3 functional outcome scores following surgical treatment for a PHF.

Our hypothesis that higher levels of neuroticism correlate with worse functional outcomes after a surgically treated PHF has been demonstrated by our results. Results show correlations between neuroticism and different functional outcomes. This finding also aligns with existing studies that assess the influence of neuroticism on physical functioning after joint surgery. Psychological factors^32^^,^^33^ are already known to play an important role after knee or hip replacement. Our findings now indicate that these also apply to the shoulder joint.

In orthopedic trauma surgery, there has been a shift from focusing solely on the physical factors that influence surgical outcomes to considering mental and social factors as well.^23^^,^^28^ While we believe this shift is necessary to better understand outcome, most studies still focus on overall health and pain rather than specific mental factors like neuroticism.^5^^,^^6^^,^^15^^,^^24^^,^^32^ As more knowledge about mental and social influences becomes available, it is now important to take further steps and collaborate with the medical psychologist. Studies like the COPE trial^9^ in which patients are given online cognitive behavioral therapy after fracture care are being performed; however, more research is needed to render a broader understanding of how to optimize postoperative care and functional results.

### Limitations

Firstly, for this study we used a retrospective cohort for the functional outcomes. Therefore, the questionnaire to determine the level of neuroticism was not administered at the time of the fracture but at the time of follow-up or later. This might impose the idea that it can influence the results as people could potentially have different scores at different times. However, the personality trait neuroticism is proven to be stable over time^7^ and if this trait is subjective to change this change mostly takes place in young adulthood (20-40 years).^29^ An age our patients already surpassed. We recognize that the impact of a negative life event can influence how someone's character is expressed or displayed and therefore may seem as if their personality is changing but the fundamental character remains unchanged. Therefore, we believe that the timing of administering the test did not influence our results.

Secondly, the median follow-up time was 12 months but not every patient had their yearly follow-up exactly 12 months after surgery to assess their functional outcomes. Thirteen patients had a follow-up of more than 2 years. This variation in follow-up time could influence the results, as a longer period might allow more time for adjustment to the new shoulder after surgery. However, if we analyze the groups above (n = 13) and under (n = 51) 1 year of follow-up there is no difference in mean OSS, qDASH, or CSS for fracture side score, meaning that the difference in follow-up time probably had no influence on the functional outcomes in our study.

Thirdly, although the CSS for fracture side is correlated with the level of neuroticism, the CSS difference score between the 2 arms is not correlated. This might be explained by the fact that these patients also perceive the nonaffected side as imperfect, since they generally have a more negative outlook on life and perceive their health as worse than those without neuroticism.^20^^,^^26^

Lastly, due to the inclusion period from 2013 to 2023, various surgical techniques were employed with different surgeons and different implants were being used, reflecting advancement in technique and materials over this time. This variability could in theory influence on the results. However, there is no correlation between the surgical year and the OSS (*P* = .227) and qDASH (*P* = .08) and therefore for these scores a relevant influence on our results by treatment variability is considered unlikely. For the CSS in the fracture side, there is a correlation with the surgical year (*P* < .001) and therefore might be of influence. However, having a longer inclusion period with various technique does suggest that our results are not limited to a specific implant but are more generalizable.

## Conclusion

A correlation between neuroticism and functional outcomes of the affected shoulder after PHF surgery was found. The correlation found in this study underscores the significance of psychological factors in the treatment and rehabilitation process of surgically managed PHFs. Orthopedic trauma surgeons should be aware of the impact of neuroticism and could screen patients for this personality trait to try and improve functional results.

Even though the correlation is there, the next step that should be considered is to see if and how these patients can be further assisted in getting better functional outcomes. Although a person's personality trait will generally stay stable,^7^ a person can be taught how to deal with the consequences of this personality trait, for example by (mindfulness based) cognitive behavioral therapy.^4^^,^^16^ Therefore, further research is needed, preferable in a prospective trial design, assessing whether psychological treatment or guidance of these patients improves their functional outcomes. Therefore, a multidisciplinary intervention study is currently being set-up as a collaborative effort between orthopedic traumasurgeons and medical psychologists.

## Disclaimers

Funding: Dr. Blaas has received a unrestricted grant from Mathys for research purposes. Dr. Derksen has received an unrestricted grant from Mathys for research purposes. Prof. Dr. van den Bekerom has received a financial contribution from Smith and Nephew to the department to support a fellowship in the field of Orthopedic Surgery.

Conflicts of interest: Leanne S. Blaas and Robert Jan Derksen have received an unrestricted educational grant from Mathys Medical Ltd. for research on proximal humerus fractures. Michel P.J. van den Bekerom has received a financial contribution from Smith and Nephew to the department for funding a fellowship in the field of Shoulder and Elbow Surgery. The other authors, their immediate families, and any research foundations with which they are affiliated have not received any financial payments or other benefits from any commercial entity related to the subject of this article.
